# Comprehensive analysis of human chorionic membrane extracts regulating mesenchymal stem cells during osteogenesis

**DOI:** 10.1111/cpr.13160

**Published:** 2021-11-28

**Authors:** Yoon Young Go, Sung‐won Chae, Jae‐Jun Song

**Affiliations:** ^1^ Department of Otorhinolaryngology‐Head and Neck Surgery Korea University Guro Hospital Seoul Republic of Korea; ^2^ Institute for Health Care Convergence Center Korea University Guro Hospital Seoul Republic of Korea

**Keywords:** bioinformatics, chorionic membrane extracts, mesenchymal stem cells, osteogenesis

## Abstract

**Objective:**

Human chorionic membrane extracts (CMEs) from placenta are known to be a natural biomaterial for bone regeneration, with their excellent osteogenic efficacy on osteoblasts. However, little is known about the regulatory mechanism involved.

**Methods and Results:**

We have shown the *in vitro* and *in vivo* bone‐forming ability of CME using human osteoblasts and bone defect animal models, suggesting that CME greatly enhances osteogenesis by providing an osteoconductive environment for the osteogenesis of osteoblasts. Proteomic analysis revealed that CME contained several osteogenesis‐related stimulators such as osteopontin, osteomodulin, Thy‐1, netrin 4, retinol‐binding protein and DJ‐1. Additionally, 23 growth factors/growth factor–related proteins were found in CME, which may trigger mitogen‐activated protein kinase (MAPK) signalling as a specific cellular signalling pathway for osteogenic differentiation. Microarray analysis showed four interaction networks (chemokine, Wnt signalling, angiogenesis and ossification), indicating the possibility that CME can promote osteogenic differentiation through a non‐canonical Wnt‐mediated CXCL signalling–dependent pathway.

**Conclusions:**

The results of this study showed the function and mechanism of action of CME during the osteogenesis of osteoblasts and highlighted a novel strategy for the use of CME as a biocompatible therapeutic material for bone regeneration.

## Graphical abstract

Go et al. report the therapeutic effects of human chorionic membrane extract during bone regeneration and its mechanism studies via *in vitro*/*in vivo* osteogenesis model and multi‐omics approaches. Their findings provide new insight into natural biomaterial and indicate the feasibility of placental tissue application for bone healing in regenerative medicine.

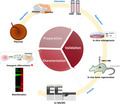

**Highlights**

- CMEs stimulate osteogenesis.
- CMEs contain osteogenic stimulators.
- Osteogenic regulators in CME induce a non‐canonical Wnt‐mediated CXCL signalling‐dependent osteogenesis

## 1 INTRODUCTION

Bone morphogenetic protein 2 (BMP2) was approved by the Food and Drug Administration (FDA) in 2004 for the regenerative stimulation of bone fractures and tooth loss in orthopaedic surgery and dentistry.^1^ However, clinical side effects were reported, including an increase in bone resorption, bone cyst formation, inflammatory responses and tumorigenesis in the bone defect area.^2^, ^3^ Additionally, BMP2‐based treatment during orthopaedic and dental procedures is expensive. Meanwhile, the target patient population is continuously increasing worldwide as people live longer.^4^ Therefore, the demand for a new alternative osteoinducer has emerged in the bone growth stimulator market, following the development of stem cell therapy using autologous bone marrow mesenchymal stem cells and the engineering of functional BMP2‐expressing human cells for bone healing.^5^, ^6^ Unfortunately, conventional recombinant BMP2 treatment retains the dominant market position in the field of orthopaedics and dentistry because alternatives with satisfactory safety and efficacy have not yet been found.

The human amniotic membrane is an attractive tissue source for regenerative medicine and has been applied in various reconstructive surgical procedures as a biological dressing since the early 20th century.^7^ Amniotic membranes have low immunogenic potential and contain bioactive factors, which allow their application for their functional properties, including anti‐inflammatory, antibacterial, antiangiogenic and proapoptotic features.^8^, ^9^, ^10^, ^11^ The best‐known applications of amniotic membrane are ocular surface reconstruction, skin applications and tissue engineering.^12^, ^13^ Recent FDA approvals for amniotic membrane‐based products such as suspensions, powder and allografts for surgical wound treatment are contributing to the growth of the amniotic membrane market.^14^, ^15^ However, the application of amniotic membranes in the human body has been restricted by a poor understanding of amniotic membrane contents. Emerging omics studies that provide collective information about the genome, metabolome and proteome now allow us to quantify the total proteins in the amniotic membrane.

Previously, we demonstrated that amniotic membrane extract (AME) and chorionic membrane extract (CME) contain different compositions of growth factors, which affect the overall osteogenic differentiation of osteoblasts.^16^ We found that CME directly affects the *in vitro* osteogenesis of human osteoblasts. We extended the study of CME for the development of an osteoinductive biomaterial in our present study. The functional properties of the chorionic membrane in osteogenesis were sequentially determined using omics approaches such as proteomics, microarray and bioinformatics. In particular, the chorionic membrane (CM) is not well understood, despite the fact that Thomas J Koob et al. showed that the CM possesses fourfold or fivefold more cytokines and growth factors than the amniotic membrane (AM).^17^ Here, we investigated the functional properties and cellular responses of the CM during the osteogenesis of human mesenchymal stem cells (hMSCs) using protein and transcriptome profiles to discover the triggered biological cellular pathway. These approaches will provide insight into this natural biomaterial and indicate the feasibility of CME application for bone growth in the field of regenerative medicine.

## 2 RESULTS

### 2.1 CME induces osteogenic differentiation of hMSCs

**FIGURE 1:**
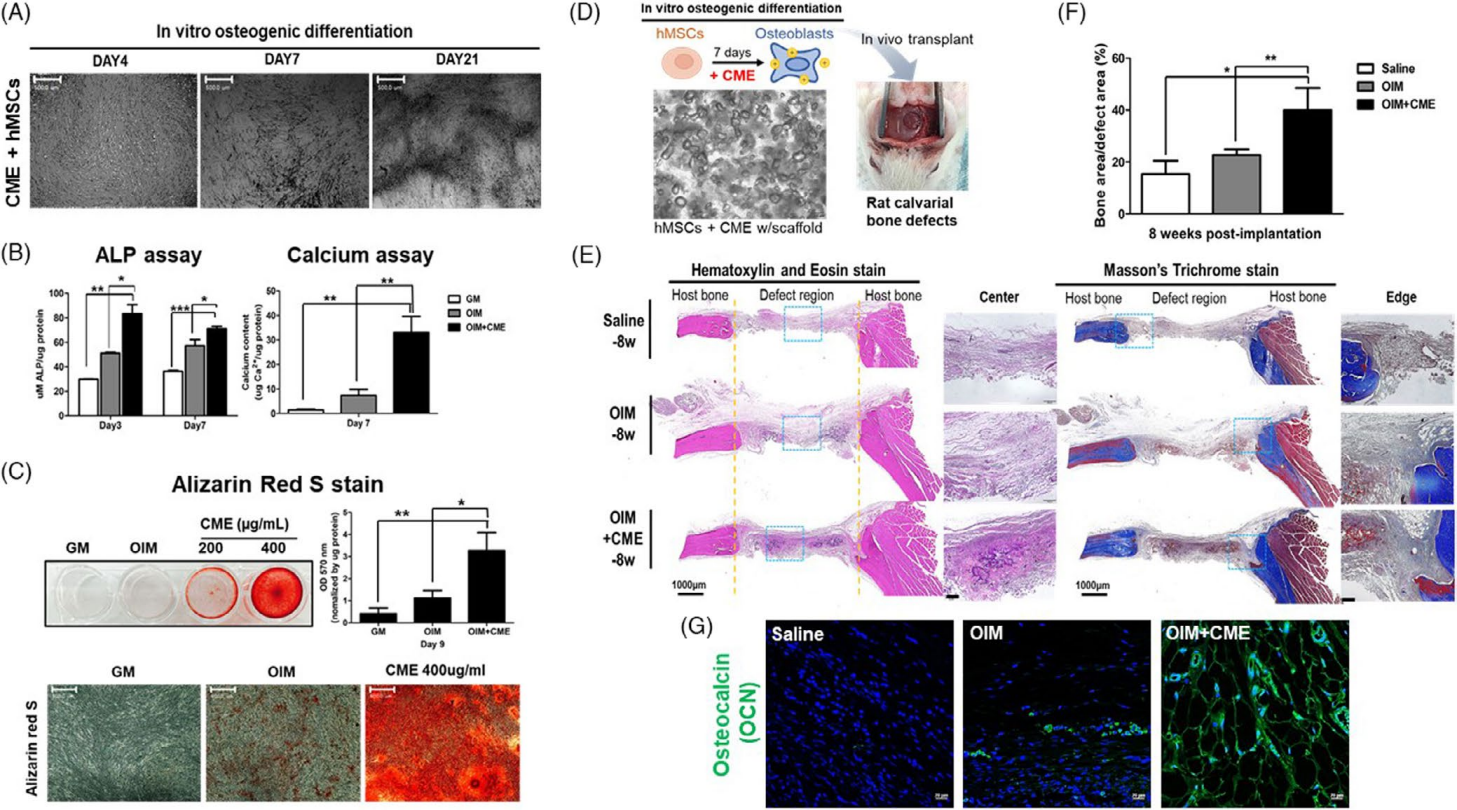
*In vitro* and *in vivo* osteogenic efficacy of CME. (A) Morphological changes in CME‐treated hMSCs were observed under a light microscope during *in vitro* osteogenesis. Scale bars, 500 μm. (B) Here, 400 μg of CME‐treated hMSCs were cultured in OIM; then, the ALP activity was determined at days 3 and 7. The calcium content was examined on Day 7. hMSCs cultured with growth medium (GM) and OIM only were used as controls. Error bars indicate the mean and SD; *p < 0.05, **p < 0.01 and ***p < 0.001 compared with control. (C) Image of Alizarin Red S‐stained hMSCs on Day 9 in GM, OIM and OIM+CME. Scale bars, 500 μm. The levels of staining of hMSCs were measured at 570 nm. Error bars indicate the mean and SD; *p < 0.05 and **p < 0.01 compared with control. (D) Schematic image of the experimental protocol applied in the rat calvarial bone defect region with hMSC‐laden scaffolds. (E) The appearance of H&E and trichrome staining of bone defect regions treated with scaffold only (saline), hMSCs (OIM) and CME‐hMSC‐laden (OIM+CME) scaffolds after 8 weeks of implantation. The yellow line indicates the boundary of the defect areas. Scale bars, 1000 μm. High‐magnification images (blue squares) were captured at the centre and edge of the calvarial bone defects. Scale bars, 500 μm. (F) The percentage of new bone area in defect region calculated with CME‐hMSCs, hMSCs and saline group. Error bars indicate the mean and SD; *p < 0.05 and **p < 0.01 compared with control. (G) Osteocalcin (green) staining of transplanted with saline, hMSCs (OIM) or CME‐treated hMSCs (OIM+CME) after 8 weeks. Scale bars, 20 μm

hMSCs were cultured in osteogenic induction medium (OIM), with or without CME supplementation, and their osteogenic efficacy was determined. CME‐treated hMSCs underwent morphological changes with mineralized nodule formation from Day 7 to Day 21 (Figure 1A and S1); high accumulation of ALP and a high calcium content were found on days 3 and 7 (Figure 1B). Analysis of the mineralization formation of the hMSCs at 9 days of culture showed a significant increase in mineralization in the CME‐treated hMSCs compared with that in the cells cultured in OIM only (Figure 1C). We next determined the bone tissue–forming ability of CME‐treated hMSCs using a rat calvarial defect model. The hMSCs were seeded onto scaffolds and cultured with or without CME for 7 days under *in vitro* OIM conditions; they were subsequently implanted into the rat calvarial defect regions (Figure 1D). Scaffold‐only groups, without cells, and hMSC‐only groups, without CME treatment, were used as controls (hereafter termed the saline and OIM groups, respectively). After 8 weeks, a histological examination was performed to determine bone tissue regeneration. The H&E and trichrome staining results showed intense calcification of bone tissue formation in the hMSC‐laden scaffolds treated with CME, compared with the saline and OIM groups (Figure 1E). The formation of new bone tissue was quantitatively represented by the percentage of bone area in the centre of the defect region; in the CME‐treated hMSC groups (OIM+CME), this was twofold higher than that in the cells cultured in only OIM (Figure 1F). Notably, transplantation of CME‐treated hMSCs clearly increased a high degree of fluorescence for bone‐specific osteocalcin (OCN) compared with saline and OIM groups (Figure 1G). These results indicated that CME treatment significantly promoted the osteogenic differentiation of hMSCs, which led to the regeneration of bone tissue.

### 2.2 CME proteins exhibit binding and catalytic activity

**FIGURE 2:**
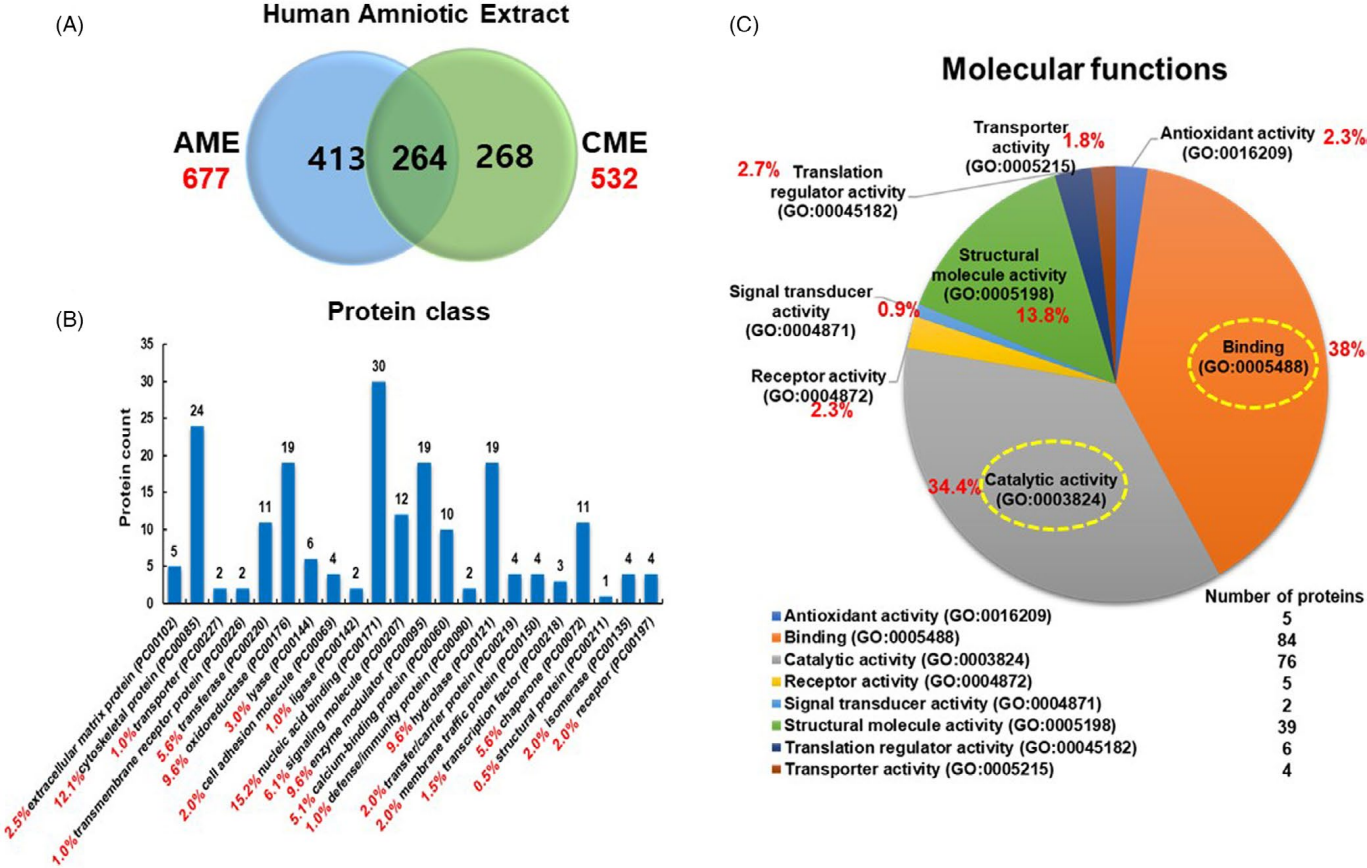
Molecular functions and protein class of CME components. (A) Venn diagram comparing the total number of proteins identified in AME and CME from human amniotic membrane. (B) PANTHER analysis of proteins contained in CME and graph showing the distribution of protein class into PANTHER protein categories. (C) Proteins categorized by molecular functions represented in this study; yellow circles indicate that most of the rated molecular functions affected by CME were binding and catalytic activity, with over 50% of proteins

The functional properties of CME for osteogenesis were confirmed, but the exact regulatory mechanism of CME in hMSCs during osteogenesis was still unknown. Previously, we showed the differential effects of AME and CME on the osteogenic commitment of hMSCs during *in vitro* osteogenesis.^16^ We first compared the weight distribution of proteins in AME and CME and confirmed the results using Coomassie blue staining (Figure S2), which indicated that similar proteins might be present in both AME and CME. If so, why does only CME demonstrate a strong osteogenic property for hMSCs? The most probable explanation is that the abundance of serum albumin (66.5 kDa), keratins (44~66 kDa) and trypsin (23.3 kDa) might have inhibited the ability to distinguish differences in the weight distribution of the protein pool between AME and CME. To further understand the differential osteogenic efficacy of AME and CME, we identified the protein pool in AME and CME using LC/MS analysis. We identified 677 and 532 proteins from AME and CME, respectively (Figure 2A). Overall, 264 proteins were present in both extracts, indicating that 50% of the proteins in CME were commonly found in AME, but 50% of proteins were specific to CME. Hence, we focused on the 268 proteins contained only in CME and analysed their protein class and molecular function using the PANTHER program. The analysis of the protein class showed the functional distribution of the CME proteins; the highest‐rated protein class was nucleic acid binding (15.2%, PC00171). Transferase (5.6%, PC00220), oxidoreductase (9.6%, PC00176), enzyme modulator (9.6%, PC00095) and hydrolase (9.6%, PC00121) made up a significant portion of the proteins associated with catalytic activity. Furthermore, signalling molecules (PC00207) were also found to make up 6.1% of the CME components (Figure 2B). The analysis also indicated that the molecular function of more than 50% of the CME proteins involved binding and catalytic activity, which are related to cell fate, such as differentiation, proliferation, migration and death (Figure 2C).

### 2.3 Growth factors in CME can trigger MAPK cellular signalling

We next performed quantitative proteomic analysis using tandem mass tag (TMT)–based quantitative mass spectrometry analysis to better understand the differential protein content of AME and CME. Among the 4905 total identified proteins, the majority were present in similar amounts in both AME and CME. However, the calculation of the 95% Gaussian fitting (−1.88 ≤ratio of Log2 CME/AME ≥1.88) revealed 66 and 46 proteins that were particularly abundant in AME and CME, respectively, as shown in Table S1.

**FIGURE 3:**
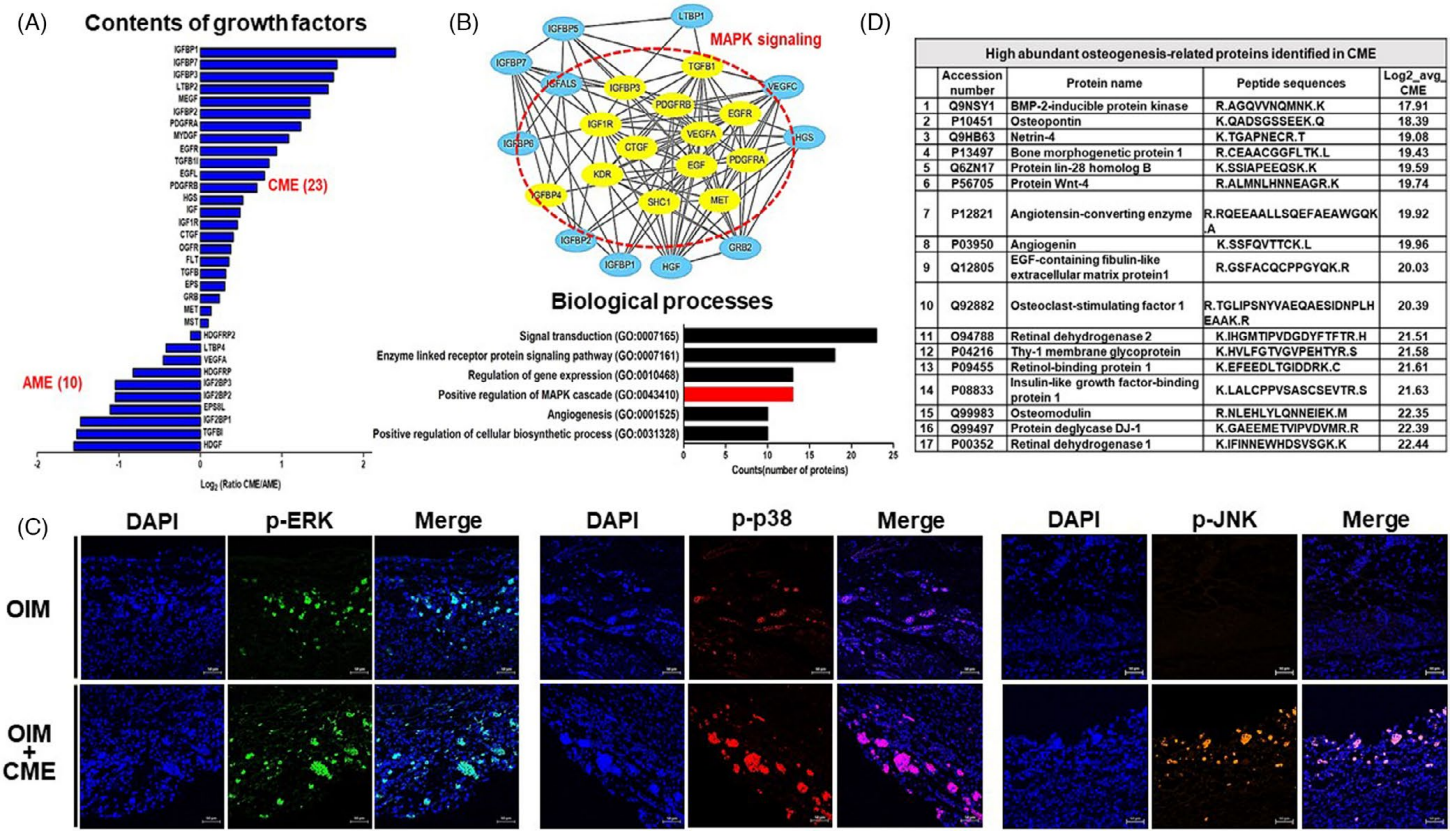
Composition of growth factors and osteogenesis‐related proteins in CME. (A) Growth factors/growth factor‐related proteins in AME and CME were subjected to proteomic analysis. Bar indicates log2 ratio of CME/AME. (B) Twenty‐three growth factors/growth factor‐related proteins of CME presented the core network of growth factors; red circle indicates MAPK signalling–related growth factors. Biological processes of GO terms analysed signal transduction, enzyme‐linked receptor protein signalling pathway, regulation of gene expression, positive regulation of MAPK cascade and angiogenesis. Bar indicates the number of each GO term–related proteins. (C) Immunofluorescence of phosphorylated ERK (p‐ERK), p38 (p‐p38) and JNK (p‐JNK) in treatment with or without CME on hMSC‐implanted bone defect model. Scale bars, 50 μm. (D) Table showing high‐abundance osteogenesis‐related proteins identified in CME. The log2 average of CME indicates the relative abundances of the m/z values from the average mass scans of total extracts

Growth factors play important roles in osteoblast proliferation and differentiation as cellular binding proteins.^18^ We specifically analysed the growth factors or growth factor–related proteins in AME and CME from the TMT analysis results. Twenty‐three and ten growth factors or related proteins were differentially identified in CME and AME, respectively (Figure 3A). CME contained twofold more growth factors than AME, including several known osteogenesis‐related growth factors such as insulin growth factor (IGF), platelet‐derived growth factor (PDGF) and connective tissue growth factor (CTGF). Moreover, STRING analysis determined that growth factors present in CME were correlated with mitogen‐activated protein kinase (MAPK) cascade signalling (Figure 3B). p38/MAPK activation plays an important role in the efficient stimulation of osteoblast differentiation during osteogenesis. Specifically, this cascade is involved in alkaline phosphatase (ALP) and osteocalcin expression in osteoblasts.^19^, ^20^ The analyses of immunofluorescence staining confirmed the increased activity of MAPK pathway (p‐ERK, p‐p38 and p‐JNK) in CME‐treated hMSCs, compared with the saline and OIM groups (Figure 3C and Figure S3). Therefore, these results imply that growth factors contained in CME could trigger the activation of the MAPK cascade during the osteogenesis of osteoblasts.

### 2.4 CME contains osteogenic stimulators

We then narrowed down the osteogenic‐related proteins from the list of those identified in the CME (Figure 3D). Consistent with our previous ELISA results, BMP2 was not identified as a CME component,^16^ but we did observe osteogenesis‐related proteins such as BMP2‐inducible protein kinase, BMP1, osteopontin and osteomodulin, which function in the regulation of skeletal homeostasis and development. The recently identified DJ‐1 protein is a novel osteogenic factor that promotes the osteogenesis of MSCs through the activation of FGF signalling.^21^ Additionally, recent studies showed that Thy‐1,^22^, ^23^ netrin 4^24^ and retinol‐binding protein 1^25^ are positive regulators of osteoblast differentiation. These results indicate that CME contains numerous known osteogenic stimulators or osteogenic regulatory proteins, which might be why CME has osteogenic properties in hMSCs.

### 2.5 Analysis of possible mechanisms underlying the stimulation of osteogenesis by CME

**FIGURE 4:**
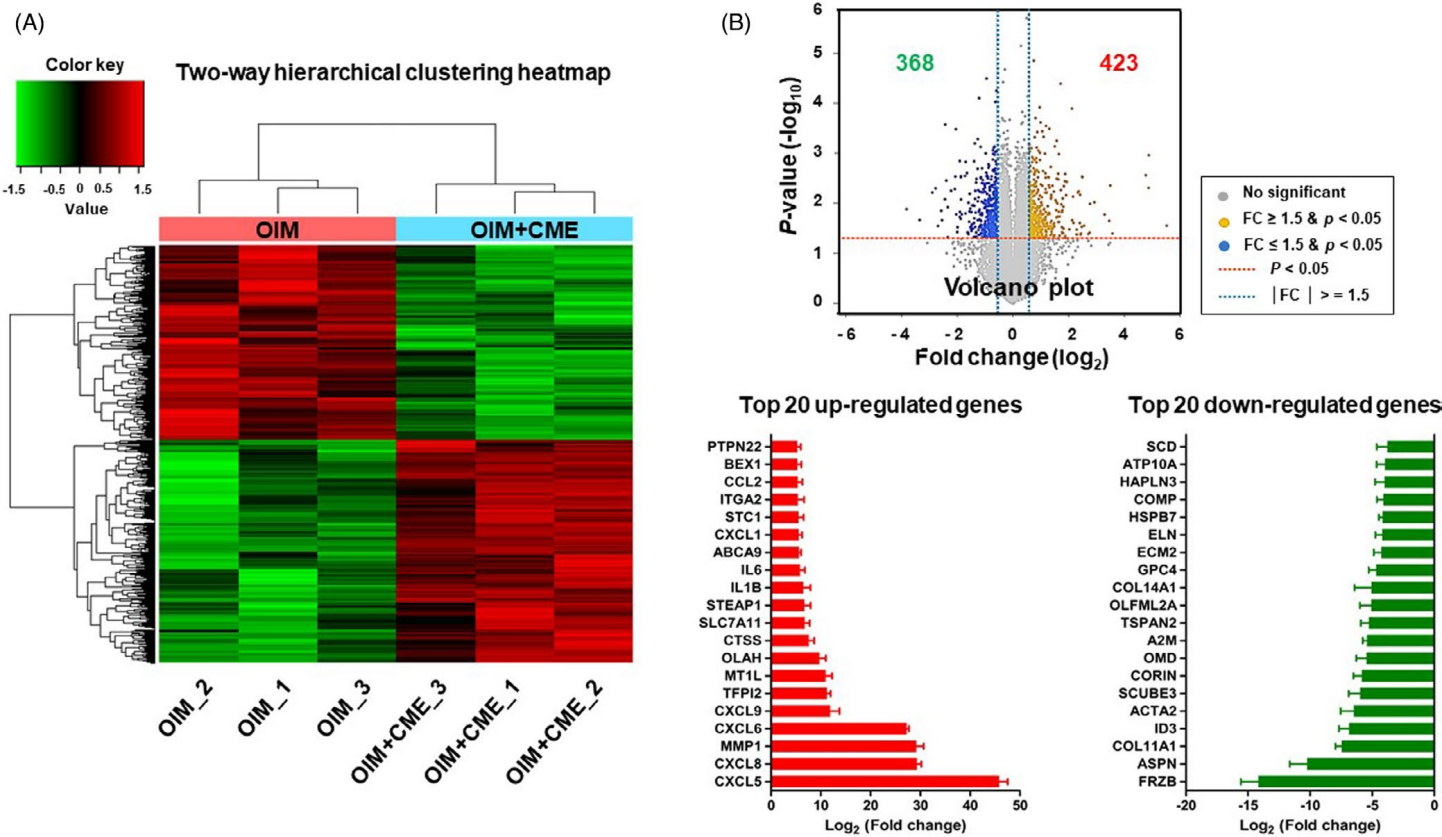
Widespread changes in gene expression profile triggered by CME (A) The heatmap clustering of microarray data shows the wide change in relative mRNA expression levels in CME‐treated hMSCs (OIM+CME) compared with untreated control cells (OIM) (1.5‐fold with *p* < 0.05). The colour bar depicts the levels of colour contrast for heatmap data using Z‐score of normalized value. (B) The Volcano plot represents the intensity of up‐ and downregulated genes in response to CME on osteogenesis of hMSCs. Each number indicate statistically significant up‐ and downregulated DEGs. Bar charts show the top list of upregulated (red) and downregulated (green) genes during osteogenesis of hMSCs with CME treatment based on microarray results (1.5‐fold with *p *< 0.05)

The next step was to analyse the alteration in the mRNA expression profile during osteogenesis in CME‐treated hMSCs using a microarray‐based approach. Microarray analysis identified 791 genes that were differentially expressed in the CME‐treated hMSCs compared with the untreated hMSCs (Figure 4A). Panel B in Figure 4B shows that 423 genes were significantly upregulated, whereas 368 genes were downregulated. The top 20 upregulated and downregulated genes are also represented. Using these data, we investigated genome‐wide changes in gene expression resulting from the hMSC’s cellular response to CME treatment during osteogenesis.

**FIGURE 5:**
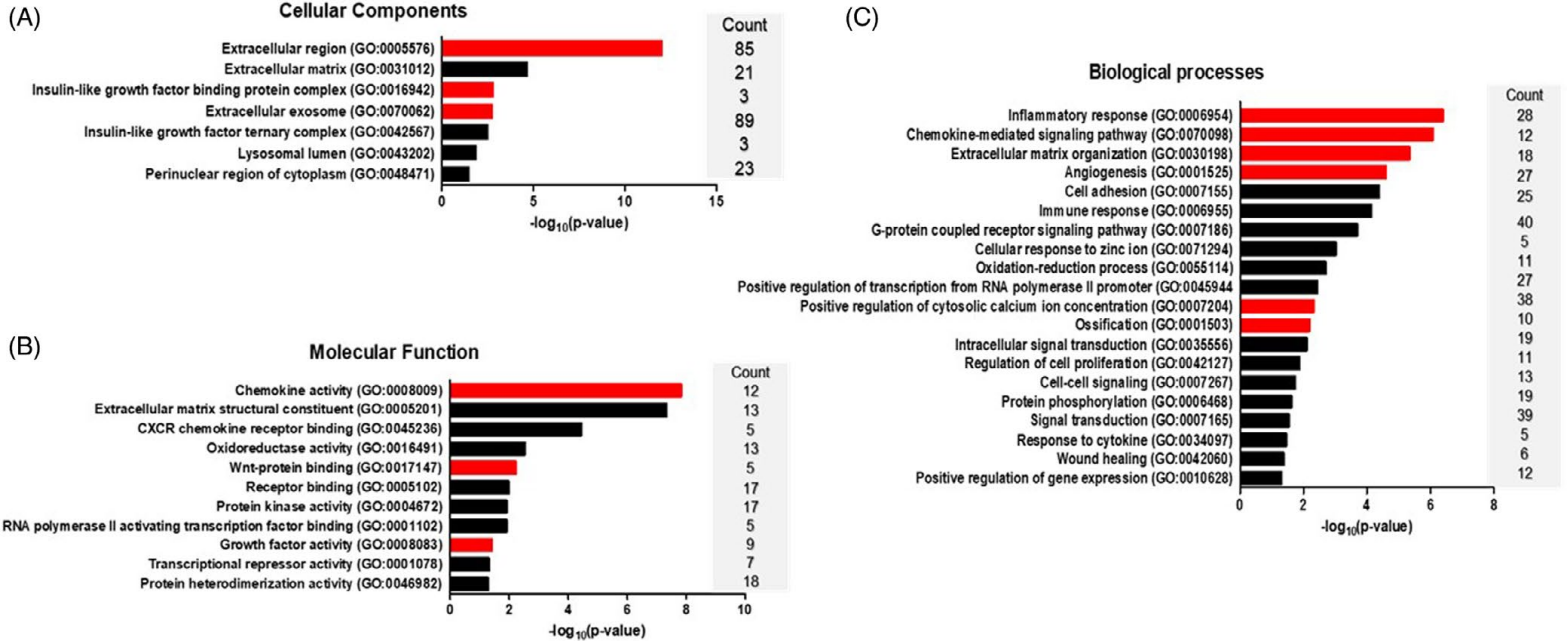
Analysis of gene ontology (GO) terms on DEGs in CME‐treated hMSCs. Graphs show significant GO terms of associated cellular components, molecular functions and biological processes from differentially regulated genes in CME‐treated hMSC (*p *< 0.05). Terms related to (A) cellular components and (B) molecular functions indicated the interaction between CME components and specific molecular signalling. (C) Key GO terms enriched in biological processes were inflammatory response, chemokine‐mediated signalling, extracellular matrix organization and angiogenesis. The red bar indicates the osteogenesis‐related GO terms

**FIGURE 6:**
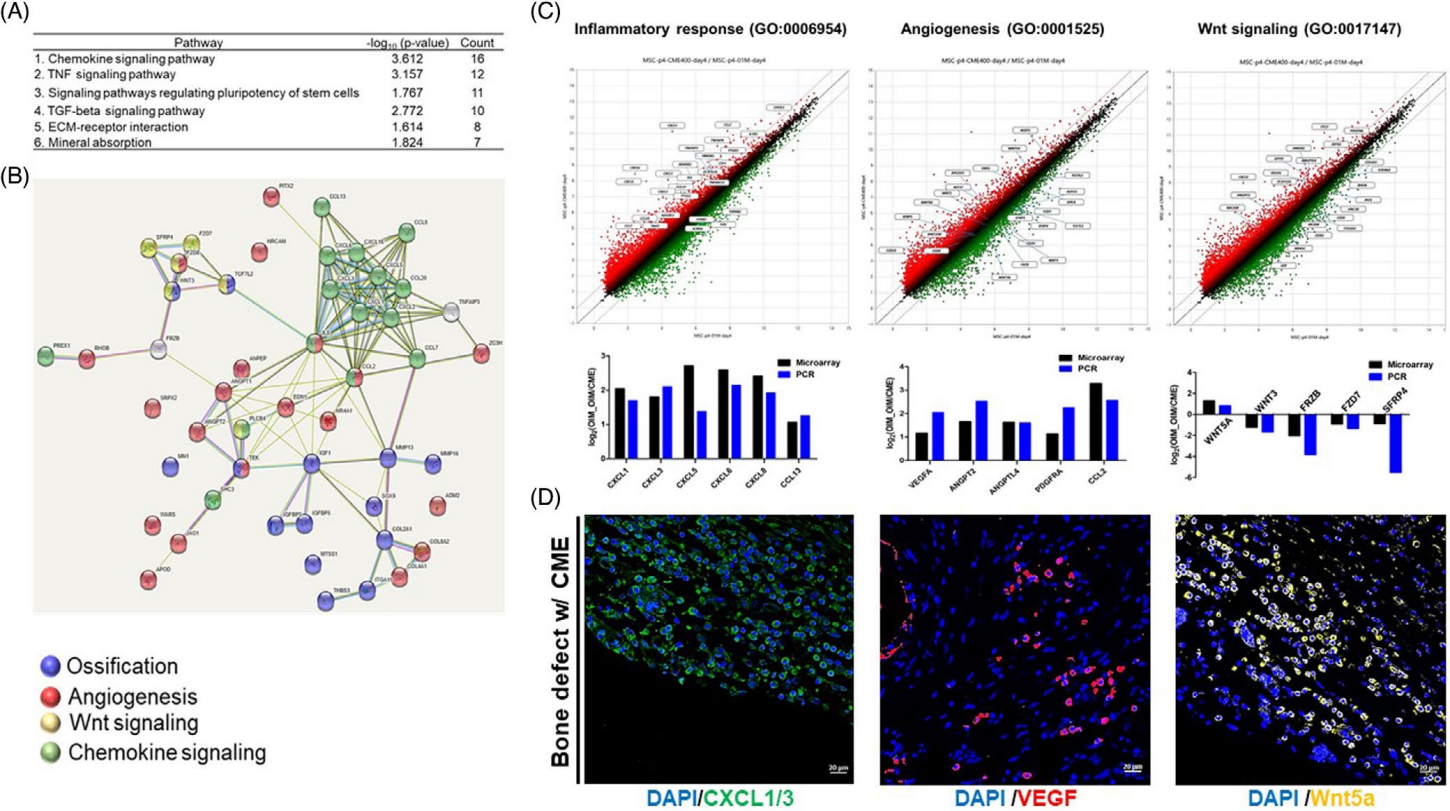
Pathway analysis based on DEGs in CME‐treated hMSCs (A) KEGG pathway analysis indicated the six most significant pathways of the differentially expressed mRNAs in CME‐treated hMSCs. The table shows the enrichment score (‐log_10_ (p‐value)) and the number of associated genes. (B) The protein‐protein interaction (PPI) network for DEGs in CME‐treated hMSCs was analysed by STRING and Cytoscape bioinformatic tools (interaction confidence >0.05). Different colour nodes indicate specific functional groups, including ossification (purple), angiogenesis (red), Wnt signalling (yellow) and chemokine signalling (green). (C) Expression change of microarray‐detected genes involving inflammatory response, angiogenesis and Wnt signalling was confirmed by quantitative real‐time RT‐PCR. All fold changes of PCR confirmation were consistent with those observed by microarray results. (D) Immunohistofluorescence staining for CXCL, VEGF and Wnt5 of *in vivo* bone defects implanted with CME‐hMSC–laden group after 8 weeks of implantation. Scale bars, 20 μm

Based on the finding of the 1.5‐fold higher up‐ and downregulation of genes in the CME‐treated hMSCs, we performed gene ontology enrichment analysis for the three GO categories, cellular components, molecular functions and biological processes (Figure 5). The cellular components of the insulin‐like growth factor‐binding protein complex, extracellular region and exosome in CME can stimulate the osteogenesis of osteoblasts. Furthermore, key GO terms significantly enriched in molecular function and biological processes were inflammatory response, Wnt‐protein binding, growth factor activity, chemokine‐mediated signalling, extracellular matrix organization and angiogenesis. Subsequently, KEGG pathway analysis demonstrated that differentially expressed genes (DEGs) in the CME‐treated hMSCs were enriched in six key pathways: chemokine signalling, TNF signalling, signalling pathways regulating the pluripotency of stem cells, TGF‐beta signalling, ECM‐receptor interactions and mineral absorption (Figure 6A). Functional enrichment analysis of the STRING and Cytoscape results visualized KEGG‐based gene networks by taxonomy and homology principles, suggesting four functional interaction networks involving chemokine signalling, Wnt signalling, angiogenesis and ossification (Figure 6B). Based on these results, a scatter plot of microarray data and quantitative real‐time RT‐PCR confirmed the functional network–related genes in the CME‐treated hMSCs (Figure 6C). The gene expression of inflammation‐related chemokines and angiogenesis‐related genes was upregulated in the CME‐treated hMSCs compared with the untreated control cells. Interestingly, we found that the expression levels of genes encoding non‐canonical Wnt ligands, such as Wnt5a, were upregulated in the CME‐treated hMSCs, but not those of the canonical Wnt pathway–related genes, such as Wnt3. The proteomic analysis results also identified several Wnt proteins such as Wnt5a, Wnt4 and secreted frizzled‐related protein 1 (SFRP1), in CME (Table S2), which are possible initiators of the non‐canonical Wnt signalling pathways. The expression levels of chemokines, such as CXCL3, CXCL5, CXCL6 and CXCL8, further confirmed that CME stimulated chemokine gene expression in a CME concentration–dependent manner (Figure S4). Moreover, the mRNA levels of CXCL gradually decreased when the cells were inhibited by interactions with CME‐containing growth factors on the cell surface, as confirmed by qRT‐PCR. Overall, immunofluorescence examination using CME‐treated hMSC‐transplanted bone defect animal model clearly showed an intensive signal for four key proteins involved in chemokine signalling (CXCL1/3), angiogenesis (vascular endothelial growth factor, VEGF), non‐canonical Wnt signalling (Wnt5A) and ossification (OCN) (Figure 6D, Figure 1G and Figure S5).

## 3 DISCUSSION

CME is an effective osteogenesis‐inducing biomaterial. It can be readily obtained from postnatal placenta and offers excellent efficacy for osteogenesis with simple processing to prepare extracts. Here, we confirmed the bone tissue–forming effects of CME using a critical rat calvarial defect model and described the possible mechanism underlying the osteogenic differentiation of hMSCs by CME treatment through a multi‐omics analysis.

Consistent with the *in vitro* results, the CME‐treated hMSCs effectively contributed to bone tissue repair through new bone formation. Our proteomic analysis systematically revealed that CME contained a significantly higher proportion of osteogenic‐related growth factors compared with AME, leading to MAPK signalling for osteogenesis. Though the identified proteins from AME were much more abundant than those from CME, AME could not promote the osteogenic differentiation of osteoblasts. This means that an optimal combination of effector proteins is important to stimulate osteogenesis in osteoblasts, rather than the number of proteins in the extract.

**FIGURE 7:**
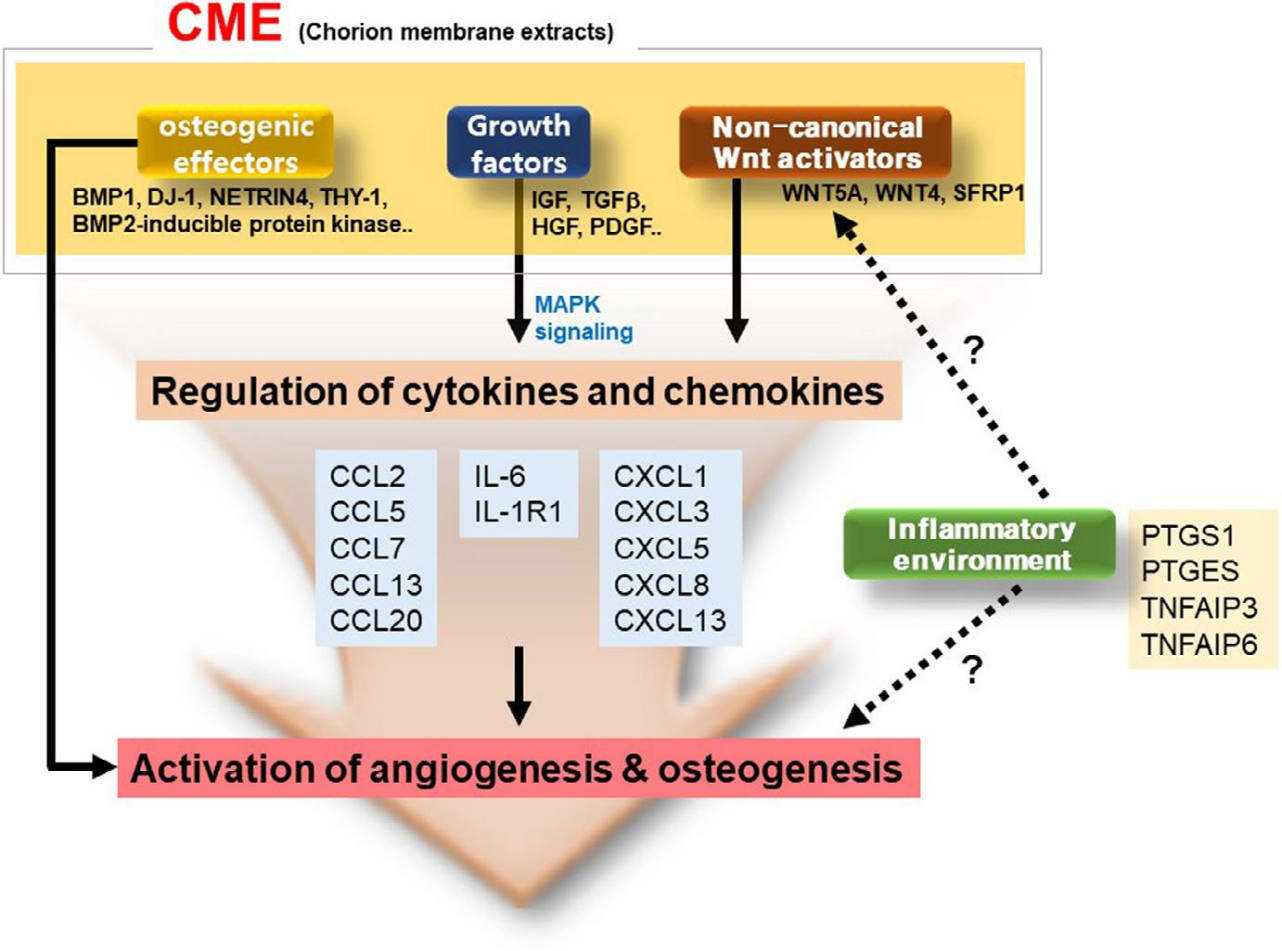
Schematic representation of the proposed regulatory mechanism underlying osteogenesis by CME on hMSCs. Proposed regulation mechanism of CME on osteogenesis of hMSC growth factors, and non‐canonical Wnt proteins in CME may trigger the activation of cytokines and chemokine signalling and subsequently promote angiogenesis and osteogenesis of hMSCs and direct osteogenic effectors in CME. Inflammatory environment caused by the activation of cytokines and chemokines may affect non‐canonical Wnt signalling and the osteogenic process, following the positive regulation of non‐canonical Wnt‐dependent CXCL‐mediated osteogenesis

Interestingly, CME not only contained several osteogenic effectors and growth factors to stimulate bone regeneration but also possessed non‐canonical Wnt activators to initiate the signalling of Wnt‐CXCL–mediated angiogenesis and osteogenesis during osteoblast differentiation (Figure 7). The pathway analysis results from microarray data also showed that a non‐canonical Wnt pathway is involved in the osteogenesis of CME‐treated hMSCs. Canonical Wnt ligands stabilize beta‐catenin via a cascade of intracellular events, facilitating its transport to the nuclei where it binds Lef1/Tcf1 transcription factors and promotes osteoblast expansion and function.^26^ However, recent evidence suggests that non‐canonical Wnt activation by Wnt5a, rather than canonical Wnt activation by Wnt3a, stimulates the osteogenic properties of bone marrow–derived MSCs.^27^ Another study showed that non‐canonical Wnt signalling induces CXC chemokines, which promote angiogenesis and osteogenesis in the early phase of bone repair.^28^ Therefore, we propose a regulation mechanism in which Wnt proteins, such as Wnt5a, Wnt4 and SFRP1, in CME initiate a non‐canonical Wnt pathway and promote the osteogenesis of hMSCs in a CXCL signalling‐dependent manner (Figure 7). The exogenous CME‐treated hMSCs of the implant could be responsible for the regulation of the cytokines and chemokines that subsequently facilitated the activation of angiogenesis and osteogenesis. The detection of Wnt5a, CXCL1/3, VEGF and osteocalcin staining in the bone defects implanted with CME‐treated hMSCs could support our hypothesis of Wnt‐CXCL–mediated angiogenesis and osteogenesis. Additionally, the CME‐treated hMSCs induced an increase in inflammatory cytokines, such as tumour necrosis factors (TNFs), interleukin‐6 (IL‐6) and prostaglandin‐endoperoxide synthase 1 (PTGS 1), in our microarray results. Croes and Albanesse et al. demonstrated that the inflammatory response and related factors are able to trigger osteogenesis and calcification via non‐canonical Wnt signalling.^2^, ^29^ However, controversial studies also reported that inflammation inhibits the osteogenesis of bone marrow mesenchymal stem cells.^30^ Liu et al. showed that the inflammatory microenvironment disturbed the non‐canonical Wnt pathway, which subsequently led to the inhibition of osteogenesis through the increase in β‐catenin in cells.^31^ Therefore, there is still conflict regarding the hypothesis that the CME‐promoted inflammatory response stimulates the osteogenesis of hMSCs via non‐canonical Wnt signalling (Figure 7).

Typically, 25–40 g of chorionic membrane (CM) tissue can be obtained from one human placenta; these amounts of CM tissue produce 63–100 mg of extract. The maximum amount of 100 mg indicates that 250 times the usage frequency can be applied for the *in vitro* osteogenesis of 2 × 10^5^ osteoblast cells because 400 µg of CME is optimal for osteogenesis in hMSCs. CME can offer a cost‐effective strategy to stimulate the osteogenesis of osteoblasts compared with recombinant BMP2 when critical bone defects are clinically treated in humans. However, CME has numerous complexities, including nucleic acids (DNA and RNA), keratins, enzymes and cell structure proteins, which is a main weakness in the development of CME as a therapeutic agent for bone regeneration in clinical settings. However, here, we indicate that CME contains an optimal combination of proteins, including several osteogenic effectors, growth factors and specific initiators, to stimulate the osteogenic differentiation of human osteoblasts (Figure 7). This study will contribute to the understanding of the regulatory mechanism of the osteogenesis of human osteoblasts and provide clues to seek new effective substances or develop new drugs for bone formation in humans.

Thus, our study demonstrates that CME‐treated hMSCs promote the repair of bone defects through the regeneration of neobone tissue in the centre and edge areas of the defect region. The proteomic analysis determined the osteogenesis‐related growth factors exclusively enriched in CME, which may trigger MAPK signalling for the osteogenesis of hMSCs. We found known osteogenesis‐related proteins, such as osteomodulin, DJ‐1, Thy‐1 and netrin 4. To investigate a novel osteogenic effect in CME, future studies need to evaluate the osteogenic ability of the 268 proteins specific to CME. To our knowledge, this study is the first investigation of the specific cellular mechanism of osteoblasts when hMSCs were treated with CME during osteogenesis. Four interaction networks (chemokine and Wnt signalling, angiogenesis and ossification) are involved in the osteogenic differentiation of CME‐treated hMSCs, suggesting that CME can stimulate the osteogenesis of hMSCs through a non‐canonical Wnt‐mediated CXCL signalling–dependent pathway. These data allow the further development of CME as a clinically available therapeutic agent to regenerate new bone in defect areas.

## 4 MATERIALS AND METHODS

### 4.1 Preparation of human CME

Human chorionic membrane (CM) was obtained from the Korea University Guro Hospital (Seoul, Korea) with the approval of the Institutional Review Board (2016GRO0141). The CME preparation procedures applied in our previous study were followed.^16^, ^32^ Briefly, the translucent amniotic membrane was separated from the amniotic membrane tissue, and the remaining tissue was used as the CM. Washed CM was sliced into small pieces, physically mashed using a homogenizer at 4°C and added to PBS at a 1:1 ratio of weight (g):volume (ml). The homogenized CM was left on ice for 1 h to allow the release of biological substances from the CM tissue and then centrifuged at 4°C. Supernatants were filtered through a 0.22‐µm syringe filter (Corning); the protein concentration was measured using a DC protein assay (Bio‐Rad). CME was stored at −80°C until use.

### 4.2 Cell culture and *in vitro* osteogenic differentiation

Human bone marrow–derived mesenchymal stem cells (hMSCs) were purchased from PromoCell (PromoCell, Heidelberg, Germany) and cultured in high glucose DMEM (HyClone) supplemented with 10% foetal bovine serum (Gibco), 1% NEAA (Lonza), 0.1% beta‐mercaptoethanol (Sigma), 1% penicillin/streptomycin (10,000 U/mL) (Gibco) and 5 mM L‐glutamine (Gibco). The hMSCs were maintained at 37°C in a humidified 5% CO_2_ atmosphere.

To induce the *in vitro* osteogenic differentiation of hMSCs, the cells were seeded in 24‐well plates (density, 1 × 10^5^ cells/well) with their growth medium and cultured upon reaching 90% confluency. The growth medium was removed and then added to osteogenic induction medium (OIM) containing additional 10 nM dexamethasone (Sigma), 0.2 mM ascorbic acid (Sigma) and 10 mM β‐glycerol phosphate (Sigma) in the growth medium. OIM was changed every 2–3 days during the *in vitro* osteogenesis of hMSCs.

### 4.3 Bone regeneration in a rat calvarial defect model

All animal experimental protocols were approved by the Institutional Animal Care and Use Committee (IACUC) of Korea University (KOREA‐2016–0199). All animal use followed the animal ethics and welfare standards according to the IACUC guidelines. To prepare the critical calvarial bone defect model, 8‐week‐old Sprague Dawley rats were anaesthetized; 2‐ to 3‐cm sagittal incisions were made in the frontal and parietal skull bones. A circular (6‐mm diameter) critical defect was created (1 defect per rat) using a trephine bur. The full thickness of the calvarial bone was removed; the Protinet scaffold (DaNAgreen), with or without cells, was immediately placed on the defect. The rats were divided into four groups: (a) defect only, (b) scaffold only, (c) hMSCs +scaffold (OIM) and (d) hMSCs +scaffold + CME (OIM/CME). hMSCs and hMSCs +CME were cultured *in vitro* on scaffolds for 7 days under OIM conditions and then implanted into the defect regions. The surgical field of the parietal skull bones was sutured. Animals were sacrificed 8 weeks after implantation.

### 4.4 Histochemical analysis and immunofluorescence staining of paraffin sections

The calvarial bone defect sites were analysed using histochemical methods, as previously reported.^16^ Briefly, the defect sites were fixed in 10% formalin overnight, decalcified in 10% EDTA (pH 7.4) for 14 days and then embedded in paraffin. The paraffin‐embedded samples were sliced at 5‐μm thickness using a rotary microtome (RM2255, Leica). The tissue sections were deparaffinized and dehydrated before haematoxylin and eosin (H&E) and Masson's trichrome staining. For H&E staining, rehydrated sections were soaked in haematoxylin solution (Sigma) for 5 min and washed with tap water. They were then immediately incubated in an Eosin‐Y solution (Sigma) for 1 min and washed with tap water. For trichrome staining, the sections were first immersed in haematoxylin solution for 15 min; then, they were washed in acetic acid (1%) (Sigma) and placed in acid orange G solution (Sigma). After washing with tap water, the sections were stained with light blue for 5 min. The stained sections were imaged using an H‐filter in colour mode.

For immunohistochemical staining, the rehydrated sections were permeabilized in 0.4% Triton‐X for 5 min and washed with PBS. Subsequently, permeabilized sections were soaked in 0.3% bovine serum albumin (BSA, Sigma) and then incubated with primary antibodies overnight at 4°C. The next day, the sections were washed thrice with PBS, and secondary antibodies were applied for 1 h in the dark. After washing, the cells were mounted using Fluoroshield with DAPI (Sigma); images were captured using a Zeiss LSM 700 confocal microscope. The antibodies used are listed in Table S3.

### 4.5 Proteomic analysis

To identify the protein content of CME, liquid chromatography‐tandem mass spectrometry (LC‐MS/MS) was performed. CME samples were prepared using filter‐aided sample preparation (FASP) as described previously.^33^ Briefly, samples were denatured in 8 M urea for 2 h; then, disulphide bonds were reduced using 10 mM dithiothreitol (DTT) for 45 min. Subsequently, 30 mM iodoacetamide (IAA) was added to the samples, followed by incubation for 30 min in the dark. The samples were then digested with trypsin at 37°C overnight. Trypsinized peptides were collected by centrifugation and desalted using a C_18_ column (Millipore). Finally, peptides were eluted using 60% acetonitrile/5% ammonium hydroxide solution; the eluted samples were dried for MS analysis. The LC–MS/MS analysis was performed using electrospray ion trap mass spectrometry (ESI‐TRAP) (Thermo Fisher). Mobile phases included 99.9% water (A phase) and 99.9% acetonitrile (ACN) (B phase), with each containing 0.1% formic acid (FA); the LC gradient time was 120 min. The MS/MS spectra were analysed using MASCOT software (version 2.5.1; Matrix Science).

The differential protein content of the AME and CME was determined using tandem mass tag (TMT)–based quantitative mass spectrometry (MS), according to previous procedures.^34^, ^35^ Sample preparation for TMT‐based quantitative MS was as described above. Briefly, AME and CME were denatured with 8 M urea and then reduced using DTT at room temperature. After alkylation of reduced extracts using IAA, proteins were quantified using the Bradford protein assay. Proteins (200 μg) from each extract were digested with trypsin at 37°C for 18 h and then concentrated and desalted using a C_18_ analytical column. Samples were subsequently tagged for quantitative mass spectrometry using a TMTsixplex Reagent Kit (Thermo Fisher); TMT‐labelled samples (TMT‐126, TMT‐128 and TMT‐129 for the AME; TMT‐127, TMT‐129 and TMT‐131 for the CME) were analysed using a liquid chromatography (LC)–MS/MS system.^36^ Mobile phases included 99.9% water and 99.9% acetonitrile (ACN), with each containing 0.1% formic acid (FA); the LC gradient time was 120 min. The MS/MS spectra were analysed using the collision‐induced dissociation high‐energy collision dissociation (CID‐HCD) method and then searched using a database on SwissProt for humans. ProLucid^37^ identified peptides with a precursor mass range of 600–6000 m/z. The output data were filtered at a false‐positive rate of less than 0.01 and a false discovery rate (FDR) of 0.1%. In this manner, 99822 peptides were identified; data with more than 30% variation were excluded. The TMT ratios for the extracts were determined using the average intensities and represented as Log_2_ values. Results with a *p*‐value <0.05 and 95% Gaussian fitting were considered statistically significant. The PANTHER program^38^ was used for the classification and analysis of molecular functions and protein class in the identified CME proteins. DAVID bioinformatics was used to analyse gene ontology (GO) annotations.^39^ Pathway analysis was conducted using the STRING database.^40^

### 4.6 Microarray analysis

Human MSCs were treated with or without 400 µg/mL of CME for 4 days; total RNA was isolated and quantified using the NanoDrop ND‐2000 (Thermo Scientific). cDNA was produced from 50 to 500 ng of RNA and then fragmented and hybridized to the array for 16 h. Microarray analysis was performed using the Affymetrix Human Gene 2.0 ST array. Three sets of microarrays, including cells cultured in growth medium (GM), OIM and OIM+CME, were used to identify differentially expressed genes in the CME‐treated hMSCs. Benjamini and Hochberg^41^ used an adjusted *p*‐value <0.05. Genes that were up‐ or downregulated by 1.5‐fold were assessed for functional and pathway enrichment analyses, such as gene ontology (GO), Kyoto Encyclopedia of Genes and Genomes (KEGG) pathway and visualization of the interaction network. Data were analysed using DAVID, KEGG,^42^ STRING and Cytoscape^43^ bioinformatics resources.

### 4.7 RNA isolation and real‐time PCR

Total RNA was isolated using the RNeasy Mini Kit (Qiagen, Hilden, Germany) according to the manufacturer's instructions. RNA (1 μg) was reverse‐transcribed to 20 μl of cDNA using the PrimeScript™ 1st strand cDNA Synthesis Kit (Takara Bio). Real‐time polymerase chain reaction (RT‐PCR) was performed using the ABI Prism 7300 Detection System (Applied Biosystems), following the manufacturer's protocol. The relative mRNA levels of the genes were analysed using the 2^(−∆∆Ct)^ method and normalized to the GAPDH gene. The sequences of specific primers for RT‐PCR are listed in Table S4.

### 4.8 Statistical analysis

Experiments were performed using three extracts from each of the five donors. Student's t *t*est was used to determine statistical differences among the experimental groups. Statistically significant levels were considered as *p < 0.05, **p < 0.01 and ***p < 0.001. All assays were performed in at least three independent experiments; representative data are expressed as the mean ± standard deviation (SD).

## 5 COMPETING INTERESTS

The authors declare that they have no competing interests.

## AUTHOR CONTRIBUTIONS

Yoon Young Go, Jae‐Jun Song and Sung‐won Chae designed the experiments; Yoon Young Go performed the experiments; Yoon Young Go and Jae‐Jun Song analysed the data; and Yoon Young Go and Jae‐Jun Song wrote the manuscript.

## Supporting information

Figure S1‐S5

Table S1

Table S2

Table S3

Table S4

## Data Availability

The data of this study are available from the corresponding author upon reasonable request. **This article included several results and description from the first author's PhD dissertation.
