# Supplementary material for: Comprehensive analysis of human chorionic membrane extracts regulating mesenchymal stem cells during osteogenesis
Source: Cell Prolif. 2021 Nov 28;55(1):e13160. doi: 10.1111/cpr.13160 (PMC8780910; doi:10.1111/cpr.13160)
Supplement: Supplementary file 1 — Figure S1‐S5 [file CPR-55-e13160-s004.docx]

**Comprehensive analysis of human chorionic membrane extracts regulating of mesenchymal stem cells during osteogenesis**

Yoon Young Go^1,2^, Sung-won Chae^1^, Jae-Jun Song^1,2^*

^1^ Department of Otorhinolaryngology-Head and Neck Surgery, Korea University Guro Hospital, Seoul 08308, Republic of Korea

^2^ Institute for Health Care Convergence Center, Korea University Guro Hospital, Seoul 08308, Republic of Korea

HIGHLIGHTS

- CME stimulate osteogenesis.
- CME contain osteogenic stimulators.
- Osteogenic regulators in CME induce a non-canonical Wnt mediated CXCL signaling dependent osteogenesis.

*Correspondence: [jjsong23@gmail.com](mailto:jjsong23@gmail.com)

Running title: hCMEs regulate osteogenesis

**
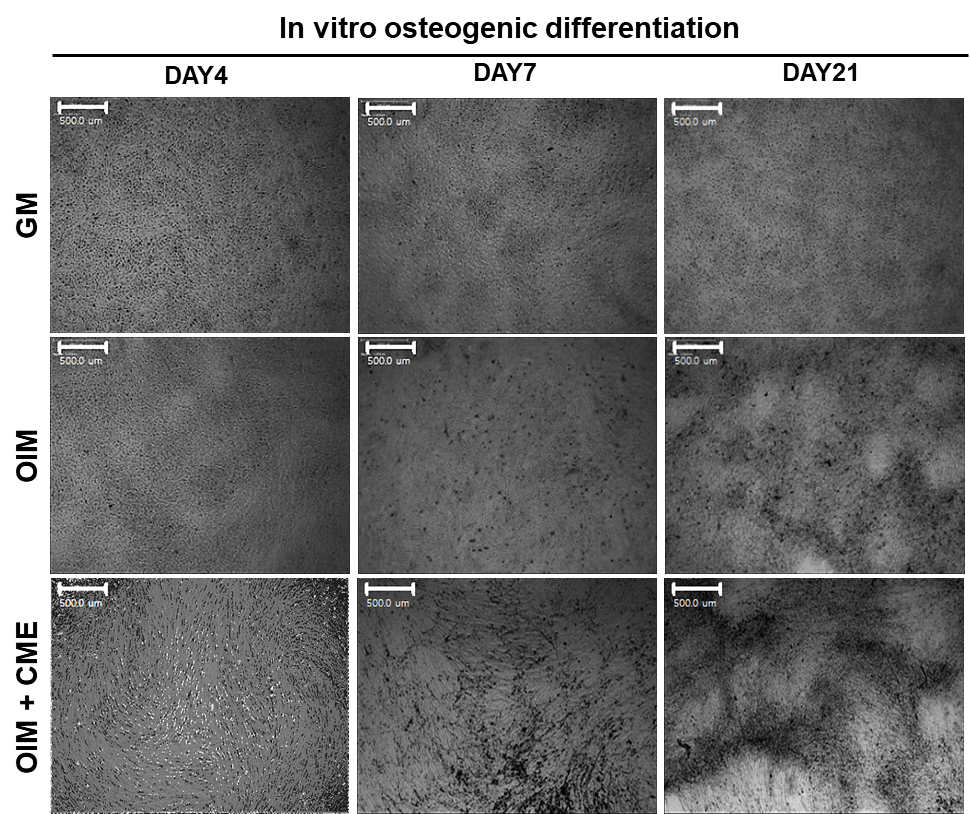
**

**Figure S1. Morphological change of CME-treated hMSCs**

Representative images of GM, OIM, and OIM+CME on hMSCs during *in vitro* osteogenesis. Images were observed under a light microscope. Scale bars, 500 μm.


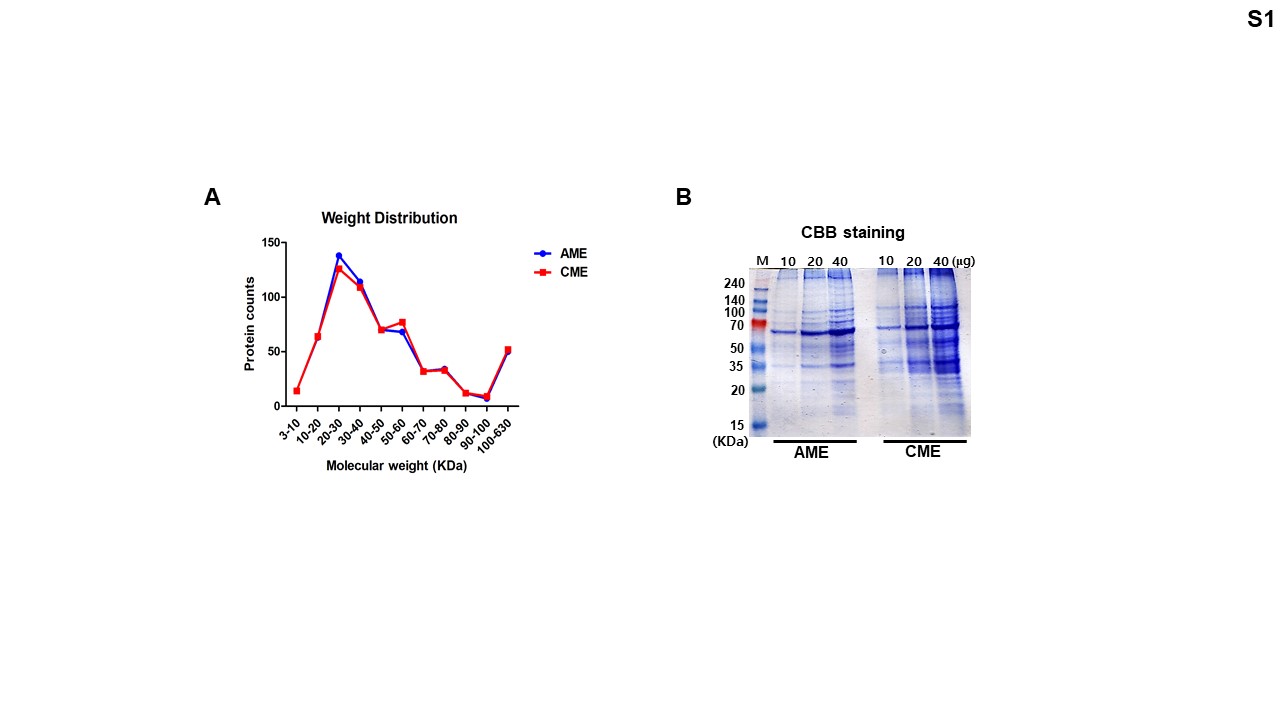


**Figure S2. Weight distribution of AME and CME**

(A) Based on the result of LC/MS analysis, the identified proteins in AME and CME were distributed by weight and are represented here. (B) AME and CME proteins separated by molecular weight on SDS page gel.


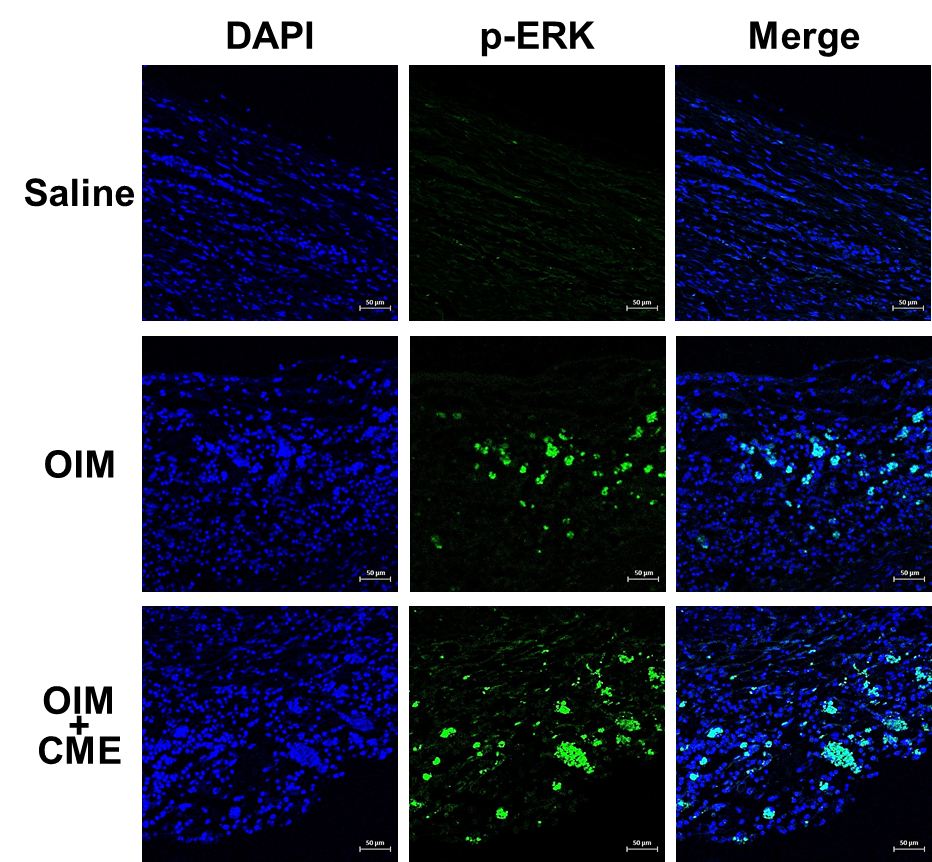


**
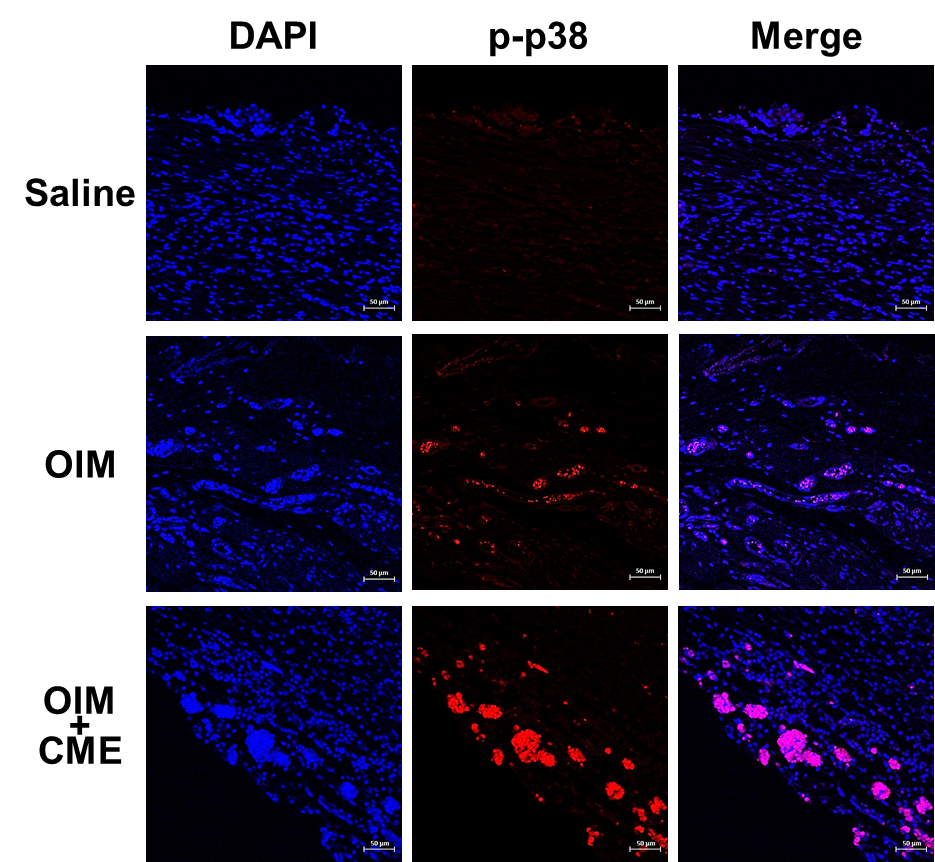
**

**
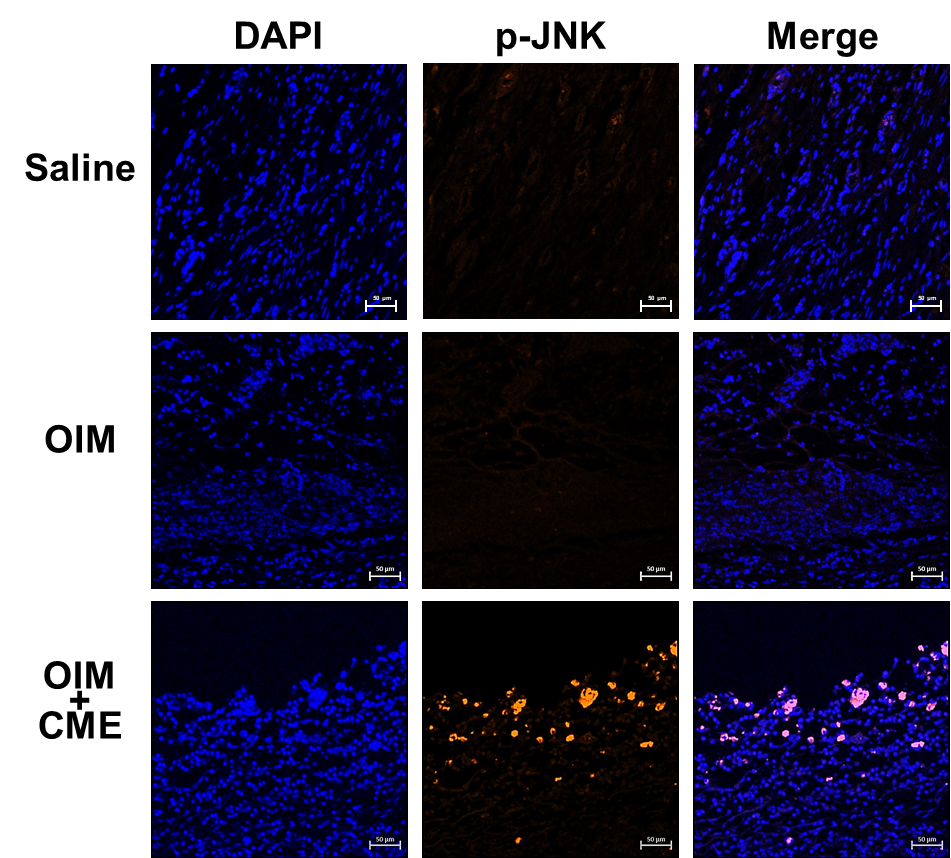
**

**Figure S3. ERK, p-38, and JNK activation in CME-treated hMSCs implanted bone defect.**

Immunofluorescence images of p-ERK, p-p38, and p-JNK in scaffold only (saline), hMSCs (OIM) and CME-hMSC-laden (OIM+CME) scaffolds after 8 weeks of implantation. Scale bars, 50 μm.


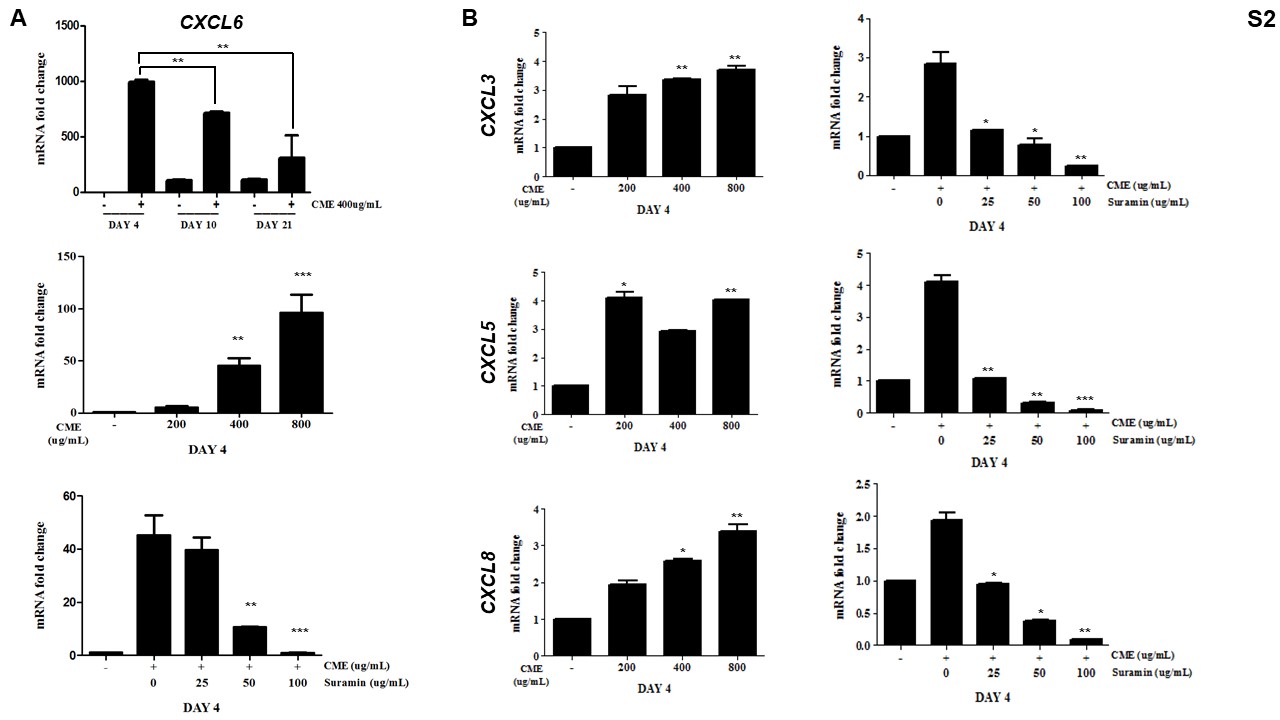


**Figure S4. CXCL expression by CME**

(A) Quantitative gene expression analysis of CXCL6 for hMSCs during *in vitro* osteogenesis in the present or absence of CME. Suramin sodium (Suramin) was used in the CME-treated hMSCs to inhibit the activity of CME-containing growth factors on cells. (B) Relative mRNA levels of CXCL3, 5, and 8 after CME treatment with or without suramin on hMSCs. Data are presented as the mean and SD of multiple repeated experiments; **p* < 0.05, ***p* < 0.01, and ****p* < 0.001 compared with control.


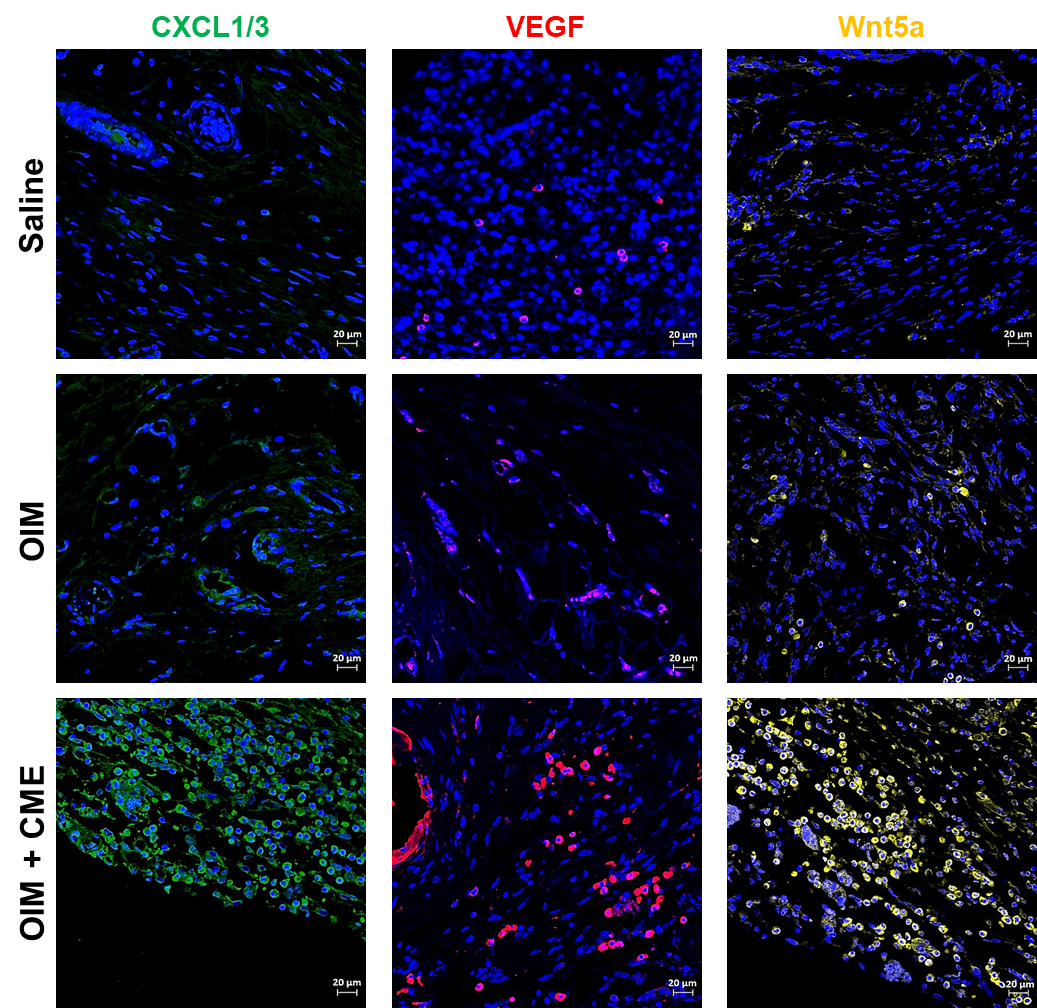


**Figure S5. Activation of chemokine, angiogenesis, and non-canonical Wnt pathway by CME**

Immunofluorescence staining of paraffin sections for CXCL, VEGF, and Wnt5 of *in vivo* bone defects implanted with saline, hMSCs (OIM) and CME-hMSCs laden groups after 8 weeks of implantation. Scale bars, 20 μm.
