# Supplementary material for: Comprehensive analysis of human chorionic membrane extracts regulating mesenchymal stem cells during osteogenesis
Source: Cell Prolif. 2021 Nov 28;55(1):e13160. doi: 10.1111/cpr.13160 (PMC8780910; doi:10.1111/cpr.13160)
Supplement: Supplementary file 3 — Table S2 [file CPR-55-e13160-s003.docx]

| **Serial number** | **Accession number** | **Protein name** | **Log2_avg_CME** |
| --- | --- | --- | --- |
| 1 | P41221 | Wnt5A | 22.39 |
| 2 | P56705 | Wnt4 | 17.91 |
| 3 | Q8N474 | SFRP1 | 19.96 |

**Supplementary information**

**Table S2. Non-canonical Wnt related proteins in CME**
