# Supplementary material for: Comprehensive analysis of human chorionic membrane extracts regulating mesenchymal stem cells during osteogenesis
Source: Cell Prolif. 2021 Nov 28;55(1):e13160. doi: 10.1111/cpr.13160 (PMC8780910; doi:10.1111/cpr.13160)
Supplement: Supplementary file 4 — Table S3 [file CPR-55-e13160-s001.docx]

**Table S3. Antibodies used in immunohistofluorescent staining**

| **Antibodies** | **Host** | **Manufacturer** | **Catalogue #** | **Category** | **Dilution** |
| --- | --- | --- | --- | --- | --- |
| Osteocalcin | Mouse | SantaCruze | SC-390877 | Primary | 1:200 |
| CXCL1/CXCL3 | Mouse | SantaCruze | SC-365870 | Primary | 1:200 |
| VEGF | Mouse | SantaCruze | SC-7269 | Primary | 1:200 |
| Wnt5A | Mouse | SantaCruze | SC-365370 | Primary | 1:200 |
| p-ERK | Rabbit | Cell Signaling | 9101 | Primary | 1:500 |
| p-p38 | Rabbit | Cell Signaling | 9211 | Primary | 1:400 |
| p-JNK | Rabbit | Cell Signaling | 9251 | Primary | 1:200 |
| Mouse IgG (488) | Goat | Invitrogen | A-11001 | Secondary | 1:300 |
| Mouse IgG (568) | Goat | Invitrogen | A-11004 | Secondary | 1:200 |
