# Supplementary material for: Comprehensive analysis of human chorionic membrane extracts regulating mesenchymal stem cells during osteogenesis
Source: Cell Prolif. 2021 Nov 28;55(1):e13160. doi: 10.1111/cpr.13160 (PMC8780910; doi:10.1111/cpr.13160)
Supplement: Supplementary file 5 — Table S4 [file CPR-55-e13160-s002.docx]

**Table S4. Sequences of oligonucleotide primers for quantitative RT-PCR.**

| Primer | Direction | Sequence |
| --- | --- | --- |
| *CXCL1* | forward | AAGTGTGAACGTGAAGTCCC |
|  | reverse | GTCACTGTTCAGCATCTTTTCG |
| *CXCL3* | forward | TGGTCACTGAACTGCGCT |
|  | reverse | ATGCGGGGTTGAGACAAG |
| *CXCL5* | forward | AGAGCTGCGTTGCGTTTGT |
|  | reverse | CTATGGCGAACACTTGCAGATTAC |
| *CXCL6* | forward | AGAGCTGCGTTGCACTTGTT |
|  | reverse | GCAGTTTACCAATCGTTTTGGGG |
| *CXCL8* | forward | ACTGAGAGTGATTGAGAGTGGAC |
|  | reverse | ACAACCCTCTGCACCCAGTT |
| *CCL13* | forward | GTCCCCAGAAGGCTGTCATC |
|  | reverse | GGACCCACTTCTCCTTTGGG |
| *VEGFA* | forward | AGGAGGAGGGCAGAATCATCA |
|  | reverse | CTCGATTGGATGGCAGTAGCT |
| *ANGPT2* | forward | GAGGCTGAGAATCAGACTGACA |
|  | reverse | TTACTGATAAACTTGCACATAACATTCT |
| *ANGPTL4* | forward | GGCGAGTTCTGGCTGGGTCT |
|  | reverse | TGG CCGTTGAGGTTGGAATG |
| *PDGFRA* | forward | CACACCTCCTCGCTGTAGTATTTA |
|  | reverse | GTTATCGGTGTAAATGTCATCCAA |
| *CCL2* | forward | TCTGTGCCTGCTGCTCATAG |
|  | reverse | TGGAATCCTGAACCCACTTC |
| *WNT5A* | forward | AGGGCTCCTACGAGAGTGCT |
|  | reverse | GACACCCCATGGCACTTG |
| *WNT3* | forward | CGCACGACTATCCTGGAC |
|  | reverse | GAGGCGCTGTCATACTTGTC |
| *FRZB* | forward | ACGGGACACTGTCAACCTCT |
|  | reverse | CGAGTCGATCCTTCCACTTC |
| *FZD7* | forward | CGCCTCTGTTCGTCTACCTC |
|  | reverse | TCATGATGGTGCGGATACGG |
| *SFRP4* | forward | TGTGTTACGAGTGGCG |
|  | reverse | GGGGGATTACTACGACTG |
| *GAPDH* | forward | TCGCCCCACTTGATTTTGG |
|  | reverse | GCAAATTCCATGGCACCGT |
